# Peste des Petits Ruminants Virus Exhibits Cell-Dependent Interferon Active Response

**DOI:** 10.3389/fcimb.2022.874936

**Published:** 2022-05-31

**Authors:** Jingyu Tang, Aoxing Tang, Hanyu Du, Nannan Jia, Jie Zhu, Chuanfeng Li, Chunchun Meng, Guangqing Liu

**Affiliations:** Shanghai Veterinary Research Institute, Chinese Academy of Agricultural Sciences, Shanghai, China

**Keywords:** Peste des petits ruminants virus, caprine endometrial epithelial cells, caprine skin fibroblasts cells, goat fibroblast cells, interferon, interferon-stimulated genes

## Abstract

Peste des petits ruminants (PPR) is an acute and highly pathogenic infectious disease caused by peste des petits ruminants virus (PPRV), which can infect goats and sheep and poses a major threat to the small ruminants industry. The innate immune response plays an important role as a line of defense against the virus. The effect of PPRV on the active innate immune response has been described in several studies, with different conclusions. We infected three goat-derived cell lines with PPRV and tested their innate immune response. PPRV proliferated in caprine endometrial epithelial cells (EECs), caprine skin fibroblasts cells (GSFs), and goat fibroblast cells (GFs), and all cells expressed interferon (IFN) by poly (I: C) stimulation. PPRV infection stimulated expression of type I and type III IFN on EECs, and expression of the latter was significantly stronger, but IFN was not stimulated in fibroblasts (GSFs and GFs). Our results suggested that the effect of PPRV on IFN was cell-type specific. Nine IFN-stimulated genes (ISGs) were detected in EECs, but only *ISG15* and *RSAD2* were significantly upregulated. The effects of PPRV on IFN and IFN-induced ISGs were cell-type specific, which advances our understanding of the innate immune response induced by PPRV and creates new possibilities for the control of PPRV infection.

## Introduction

Peste des petits ruminants virus (PPRV) belongs to the genus *Morbillivirus* in the family *Paramyxoviridae* (Amarasinghe et al., 2019). It causes acute and highly contagious disease in small ruminants such as goats and sheep and infects large ruminants such as camels, cattle, water buffalo, and various wild animals with varying morbidity and mortality (Lembo et al., 2013; Eiden et al., 2014; Zakian et al., 2016).

Interferons (IFNs) are an important group of host cell-induced antiviral cytokines that also play an important role in the regulation of adaptive immune responses (Kumar et al., 2014). There are three types of IFNs (I/II/III), which differ in structure, receptor distribution, and tissue-specific biological activities, but all induce antiviral effects. Type I IFNs comprise IFN-β, 13 subtypes of IFN-α, IFN-ϵ, IFN-τ, and IFN-ω, which are mainly expressed in innate immune cells (Mesev et al., 2019). There is only one type II IFN, IFN-γ, which is usually induced by natural killer cells and T cells (Gray and Goeddel, 1982). Type III IFNs have four members, IFN-λ1, IFN-λ2, IFN-λ3, and IFN-λ4, which mainly act on the surface of epithelial cells (Kotenko et al., 2003; Sheppard et al., 2003; Hamming et al., 2013; Wells and Coyne, 2018). Type I and III IFNs share similar pathogen-sensing pathway induction and activate the expression of associated antiviral and immunomodulatory genes (Lazear et al., 2019).

Some studies have shown that PPRV infection has a weak and transient effect on the induction of IFN-β (Sanz Bernardo et al., 2017). Recent studies have focused on the effect of type I IFNs, while there are few on the effect of type III IFNs. However, the innate immune response caused by PPRV infection mainly focuses on goat peripheral blood mononuclear cells (PBMCs) and lymphocytes, which are difficult to obtain and culture and are not conducive to subsequent studies (Manjunath et al., 2017; Wani et al., 2019); Tirumurugaan et al., 2020 Like other morbilliviruses, PPRV has obvious lymphatic and epithelial tropism and it can be isolated from many organs (Stewart et al., 2014). Nevertheless, the effects of PPRV on diverse tissue-originated cell lines are less well studied. Therefore, it is necessary to compare the IFNs response of different tissue origin cell lines after PPRV infection, which will help us understand the host’s innate immune system to resist viral infection. In this study, we first select three individual tissue origin cell lines as models to study the natural immunity induced by PPRV infection.

## Materials and Methods

### Cell, Virus, and Antibodies

Caprine endometrial epithelial cells (EECs), which were kindly provided by Prof. Yaping Jin (Northwest Agriculture & Forestry University, Yangling, Shaanxi, China) were cultured in Dulbecco’s Minimal Essential Medium/Nutrient Mixture F-12 Ham’s medium (DMEM/F12, Gibco) with 10% fetal bovine serum (FBS, Gibco). Caprine skin fibroblasts cells (CSFs) purchased from the Conservation Genetics CAS Kunming Cell Bank (No. KCB201073S) were grown in DMEM containing 10% FBS. Goat fibroblast cells (GFs) were provided by our lab and grown in DMEM containing 10% FBS. All cells were cultured at 37°C with 5% CO_2_. The PPRV Nigeria/75/1 vaccine strain was propagated in modified Vero cells which stable overexpressing the goat SLAM receptor.

### Poly (I: C) Stimulation and Virus Infection

GFs, EECs, and CSFs were transfected with Lipofectamine 3000 (Invitrogen) at 0.25μg/mL, 2.5μg/mL and 5mg/mL of HMV-poly (I: C) (Invitrogen), and samples were collected at 12 and 24 h. Three species of goat cells were infected with PPRV Nigeria/75/1 vaccine strain at a multiplicity of infection (MOI) of 1 and cultured with 2% FBS. Cell samples were collected at 12, 24, 36, and 48 h post-infection (hpi). The cytopathic effects were observed daily until 120 h after PPRV inoculation. Viral titers on Vero-SLAM cells were determined as median tissue culture infective doses (TCID_50_) as described previously (Tang et al., 2021).

### Real-Time Quantitative Reverse Transcription-Polymerase Chain Reaction (qRT-PCR)

To detect the expression level of each target gene, total RNA was extracted from the cell samples using TRIzol reagent and transcribed to cDNA according to the HiScriptIII RT SuperMix for qPCR kit (Vazyme). The qRT-PCR was performed using Novozymes Ultra SYBR Mixture (Vazyme). The gene expression of target mRNA was normalized to endogenous glyceraldehyde 3-phosphate dehydrogenase, and the comparative cycle threshold (ΔΔC_T_) method was used for relative quantifications. The corresponding primers for qRT-PCR are listed in **Table 1**.

**Table 1:** The primers for qRT-PCR.

Names	Primers (5’-3’)
GAPDH-qF(goat)	TGGAGAAACCTGCCAAGTATG
GAPDH-qR(goat)	TGAGTGTCGCTGTTGAAGTC
PPRV-N-qF	GTTATCATAGTCCCCATTCCCG
PPRV-N-qR	CTCCACGAACAAAGATAACATGC
IFNα-qF(goat) IFNα-qR(goat)	TCTTATTCCCCCAGGAGGCG GAGCTGCTGATCCAGTGCAG
IFNβ-qF(goat)	TGCCTCCTCCAGATGGTTCTCC
IFNβ-qR(goat)	TGCTGTGCTTGCTTCATCTCCTC
IFNλ3-qF(goat)	TGAGCAGGACAGGAGCAGTTCC
IFNλ3-qR(goat)	GAGCGAGTCTTCAAAGGCATCCC
ISG15-qF(goat)	CAATGTGCCTGCTTTCCAG
ISG15-qR(goat)	ACCCTTGTCGTTCCTCACC
RSAD2-qF(goat)	CCACTCCCACCAGCGTCAATTAC
RSAD2-qR(goat)	CTTCCTTCAGCATCAGCAGACCTC
TRIM25-qF(goat)	CACCGACCTGGAGAACAAGTTGAG
TRIM25-qR(goat)	TCCTGCTGCCTGGCTCTCAC
GBP2-qF(goat)	GGCTCTACCGCACAGGCAAATC
GBP2-qR(goat)	GAGGCACACACCACATCCAGATG
IFIT1-qF(goat)	AAGGCGTTCTGGATGAAGCTCTTC
IFIT1-qR(goat)	GCCCTATCTGGTGATGCTGGAAAG
Mx1-qF(goat)	TGGTGGTGGTCCCTGCTAACG
Mx1-qR(goat)	ACCTTGTCTTCTGTGCCTTTGTCC
ISG20-qF(goat)	TCCGTGCTGTACGACAAGTTCATC
ISG20-qR(goat)	CTCCATGTTCCGAGCTGTGATTCC
IFIT5-qF(goat)	GGCGGCTGAATGCTATGAGAAGG
IFIT5-qR(goat)	CATCCTGCTCCATTTCCTCACTGC
OASL-qF(goat)	GGGAAGCCAGCAACCACAACC
OASL-qR(goat)	GAATGGTGAAGGTGACAGCAGAGG

### Western Blotting

Cell samples were lysed with RIPA lysis buffer. Equal amounts of total protein were separated by 12% sodium dodecyl sulfate-polyacrylamide gel electrophoresis and subsequently transferred to nitrocellulose membranes (GE Healthcare) using a semi-dry transfer cell (Bio-Rad Laboratories). Membranes were incubated with the following primary antibodies: mouse anti-β-actin monoclonal antibody (1:1,000; Kangwei) and mouse anti-N polyclonal antibody (1:500; prepared in our laboratory) overnight at 4°C after being blocked with 5% non-fat milk in Tris Buffer Saline with Tween (TBST) buffer for 2 h at 37°C. After being washed three times with TBST buffer, the membranes were incubated with a specific peroxidase-conjugated goat anti-mouse secondary antibody for 1 h at room temperature. After being washed three times with TBST buffer, protein bands were analyzed by enhanced chemiluminescence (Thermo Fisher Scientific) using an automated chemiluminescence imager (Tanon).

### Statistical Analysis

All the results were representative of three independent experiments. The statistical significance of differences between groups was analyzed using one-way analysis of variance in GraphPad Prism 8. *P* <0.05 was considered statistically significant.

## Results

### PPRV Can Proliferate in Three Types of Goat Cells

**Figure 1:**
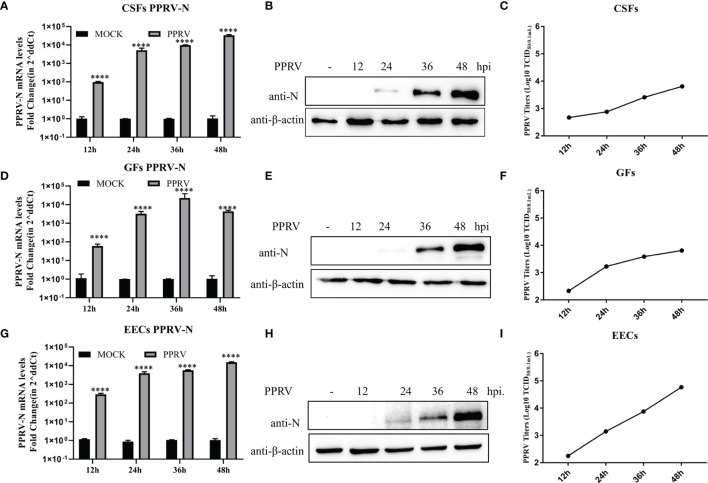
PPRV proliferated in three types of goat cells. **(A–C)** Nucleoprotein mRNA, protein levels and viral titers in PPRV-infected CSFs were determined. **(D–F)** Nucleoprotein mRNA, protein levels, and viral titers in PPRV-infected GFs GFs were determined. **(G, H)** Nucleoprotein mRNA and protein levels and viral titers in PPRV-Infected EECs were determined. ****P < 0.0001.

CSFs, GFs, and EECs were infected with PPRV (MOI)=1, and *N* gene expression and viral titers were detected at 12, 24, 36, and 48 hpi. The qRT-PCR showed that the *N* gene mRNA levels in CSFs and EECs increased gradually from 12 to 48 hpi (**Figures 1A, G**). In GFs, *N* gene mRNA levels peaked at 36 h after infection and then decreased (**Figure 1D**). Western blotting results showed that N protein expression was not detected in the three cell lines at 12 h after PPRV infection, but began to be detected at 24hpi, and then gradually increased (**Figures 1B, E, H**). After CSFs, GFs and EECs were infected with PPRV for 12, 24, 36 and48 h, the viral titers in the supernatant increased gradually (**Figures 1C, F, I**). In addition, cytopathic effects such as rounding and shedding of three kinds of cells gradually appeared after PPRV inoculation 48 h (**Supplementary 1**). These results indicated that PPRV proliferates in three goat-derived cells.

### Poly (I: C) Induces the Expression of Type I and Type III IFNs in Epithelial Cells and Fibroblasts

**Figure 2:**
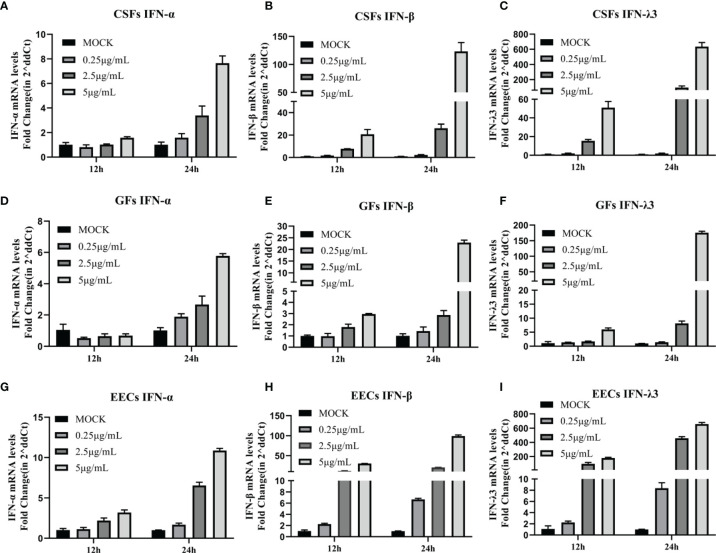
Poly (I: C) induced expression of type I and type III IFNs in epithelial cells and fibroblasts. **(A–C)** CSFs were stimulated with poly (I: C) for 12 and 24 h, and expression of IFN-α, IFN-β, and IFN-λ3 was measured by RT-PCR. **(D–F)** GFs were stimulated with poly (I: C) for 12 and 24 h, and IFN-α, IFN-β, and IFN-λ3 expression was measured by RT-PCR. **(G–I)** EECs were stimulated with poly (I: C) for 12 and 24 h, and expression of IFN-α, IFN-β, and IFN-λ3 was measured by RT-PCR.

It has been demonstrated that stimulation of GFs with the synthetic dsRNA viral ligand poly (I: C) induced effective innate immunity (Zhu et al., 2019). However, it is unclear whether poly (I: C) has the same effect on goat-derived epithelial cells and other fibroblasts for innate immunity. As part of a preliminary study, we treated EECs, GFs, and CSFs with multiple doses of poly (I: C) and collected samples at 12 and 24 h to detect the expression of type I IFNs (IFN-α and IFN-β) and type III IFNs (IFN-λ3) IFNs. Poly (I: C) in a dose-dependent manner, increased the expression of IFN-α, IFN-β, and IFN-λ3 mRNA in the three cell lines, reaching the highest level after 24 h of stimulation at 5 μg/mL (**Figures 2A–I**). IFN-α mRNA level was slightly increased and IFN-λ3 mRNA level was most significantly increased (**Figures 2A–I**). The results indicated that poly (I: C) induced an effective innate immune response in the three goat-derived cell lines.

### PPRV Induces Innate Immunity in EECs but Not CSFs and GFs

**Figure 3:**
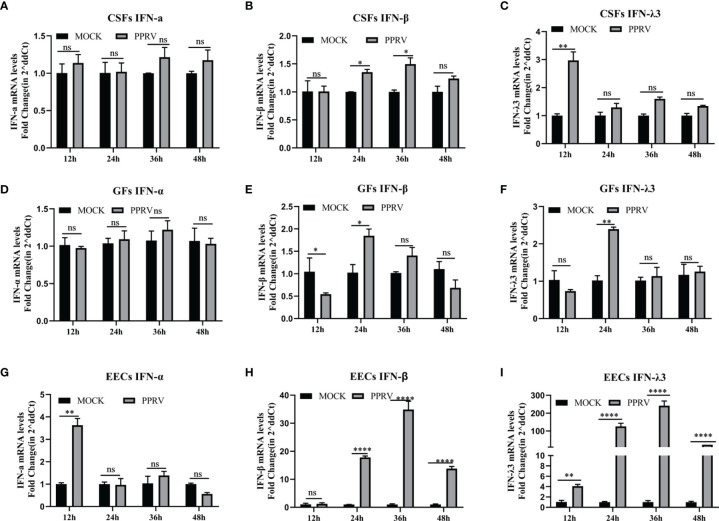
PPRV induces innate immunity in EECs, but not CSFs and GFs. **(A–C)** CSFs were infected with PPRV for 12-48 h, and expression of IFN-α, IFN-β, and IFN-λ3 was measured by RT-PCR. **(D–F)** GFs were infected with PPRV for 12-48 h, and expression of IFN-α, IFN-β, and IFN-λ3 was measured by RT-PCR. **(G–I)** EECs were infected with PPRV for 12-48 h, and expression of IFN-α, IFN-β, and IFN-λ3 was measured by RT-PCR. Comparisons between groups were calculated using two-way ANOVA in GraphPad Prism 8. *P < 0.05; **P < 0.01; ***P < 0.001; ****P < 0.0001; ns, no significant difference.

To determine whether PPRV stimulated IFNs production, GSFs, GFs, and EECs were infected with PPRV for up to 48 h. In both types of fibroblasts, GSFs and GFS, PPRV did not alter the expression of IFN-α, and the levels of IFN-β and IFN-λ3 mRNA were only slightly increased compared with those in the untreated cells (**Figures 3A–F**). In EECs, IFN-α mRNA levels were elevated at 12 h and subsequently were not significantly different from those in the untreated group. However, IFN-β and IFN-λ3 mRNA levels were significantly elevated at 24 h after PPRV infection, peaked at 36 h, and then decreased (**Figures 3G–I**). The results suggested that different cells could produce different innate immune responses after PPRV infection.

### PPRV Induces Upregulation of *RSAD2* and *ISG15* on EEC Cells

**Figure 4:**
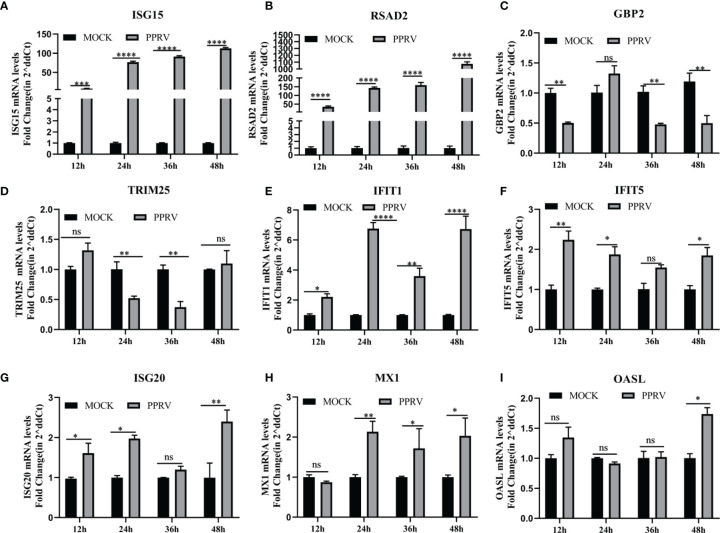
PPRV induced upregulation of *RSAD2* and *ISG15* on EEC cells. **(A–I)** EECs were infected with PPRV for 12-48 h, and expression of *ISG15*, *RSAD2*, *GBP2*, *TRIM25*, *IFIT1*, *IFIT5*, *ISG20*, *MX1* and *OASL* was measured by RT-PCR. Comparisons between groups were calculated using two-way ANOVA in GraphPad Prism 8. *P < 0.05; **P < 0.01; ***P < 0.001; ****P < 0.0001; ns, no significant difference..

We then investigated whether PPRV regulated IFN-induced downstream ISGs on EECs. Nine ISGs mRNA levels were analyzed by qRT-PCR after PPRV infection, including *GBP2*, *ISG15*, *ISG20*, *IFIT1*, *IFIT5*, *MX1*, *OASL*, *RASD2*, and *TRIM25*. The results showed that mRNA levels of *ISG15* and *RSAD2* were significantly upregulated over time, with a maximum of about 100-fold and 1,000-fold, respectively (**Figures 4A, B**). *GBP2* was downregulated at 12, 36, and 48 h (**Figure 4C**), and *TRIM25* was downregulated at 24 and 36 h (**Figure 4D**). IFIT1 and MX1 to be consistently and significantly upregulated (**Figures 4E, H**). And other ISGs were irregularly upregulated or did not change significantly compared to the untreated group (**Figures 4F, G, I**). The results suggested that there was a difference in the response of ISGs to PPRV infection of EECs.

## Discussion

Currently, Vero cells or some cell lines expressing PPRV receptors are mainly used for PPRV studies *in vitro*, but little is known about PPRV in goat cells (Kumar et al., 2013; Clarke et al., 2018; Begum et al., 2021). Because the natural hosts of PPRV are mainly small ruminants such as goats and sheep, goat cells should be suitable for the study of viral infection and immunity to PPRV, and only such evidence can objectively reflect the pathogenic and natural immune mechanisms of PPRV in the host. In this study, three goat cell lines (EECs, CSFs, and GFs) were selected to compare the proliferation and innate immune response after PPRV infection, all kinds of the cells can support the proliferation of PPRV and appear obvious cytopathological effects.

PPRV has a well-established lymphatic and epithelial tissue tropism (Birch et al., 2013; Pope et al., 2013). Lymphocytes played a dominant role in the resistance to PPRV infection (Qi et al., 2019; Wani et al., 2019). The cell adhesion molecule Nectin-4 has been identified as a receptor for PPRV, mediates PPRV infection of epithelial cells, and plays a major role in virus transmission (Birch et al., 2013; Prajapati et al., 2019). However, PPRV has rarely been studied in epithelial cells. Here, to investigate the immune response of goat cells infected with PPRV, we first stimulated three goat cell lines with poly (I: C) and confirmed that all these cells could effectively induce an innate immune response, showing that expression of type III IFNs was significantly higher than the expression of type I IFNs. Then, the three cell lines were infected with PPRV. PPRV stimulated expression of type I and type III IFNs only in EECs, but not in GSFs and GFs, and expression of type III IFNs was significantly higher than the expression of type I IFNs. These results are consistent with other research showing that PPRV does not stimulate IFN-β production in goat fibroblasts (Zhu et al., 2019). Chang et al. reported that IFN-λ3 was strongly induced by PPRV infection of HEK-293T cells, while transcript levels of IFN-β and IFN-λ2 were moderately induced (Chang et al., 2019). RIG-I receptor is mainly responsible for sensing its double-stranded RNA and stimulating the cell to produce IFNs after PPRV infection. The evolution of the RIG-I receptor is highly conserved, and there is no significant difference in tissue distribution. However, fibroblasts do not produce significant upregulation of IFNs after PPRV infection. We speculate that the host innate immunity is a complex cascade reaction system and poly (I: C) as a positive stimulus can induce the cell to produce IFNs, which can only indicate that the IFNs response pathway of the cell is not defective. The induction of IFNs after virus infection is much more complex than poly (I: C). Specifically, proteins encoded by the virus may interact with different proteins in the host cell to enhance or inhibit the activity of an effector molecule in the IFNs pathway, and then affect the expression level of IFNs. Different origins of fibroblasts and EECs cells may lead to significant differences in some factors interacting with viral proteins, resulting in obvious differences of IFNs responses (Qian et al., 2017).; Liu et al., 2019; Chen et al., 2021; Deng et al., 2022

IFN-λ3 is upregulated 10 times more than IFN-β after PPRV infection of EECs, which may be related to the distribution of IFNs receptors. Almost all cells express the functional type I IFNs receptor IFNAR while the expression of the type III receptor IFNLR complex is most commonly restricted to cells on mucosal and other barrier surfaces. Antiviral responses in epithelial cells are mainly mediated by type III IFNs (Lazear et al., 2015; Mesev et al., 2019). Although their receptor complexes have differences, once they bind to their respective receptors, their downstream signaling processes are virtually identical and lead to the induction of hundreds of ISGs through typical signaling pathways (Zhou et al., 2007). In the current study, *ISG15* and *RSAD2* mRNA levels were upregulated by PPRV infection. Similar results were obtained *in vitro* with PPRV live attenuated vaccine (Sungri/96) immunization, with *ISG15* significantly elevated in vaccinated goats at 5 and 14 days and in sheep at 7 days (Wani et al., 2018). Transcriptome analysis of PPRV-infected lymphocytes and monocytes has revealed significant differences in their overall gene expression profiles, with the IFNs signaling pathway (*ISG15, Mx1, Mx2, RSAD2, IFIT3*, and *IFIT5*) and RIG-I-like receptor signaling pathway activated in lymphocytes but not in monocytes (Wani et al., 2019). Another study found that *ISG15, IRF7, IFI44, RSAD2, OAS1X*, and *IRF7* were upregulated after PPRV infection in goats by analyzing the transcriptome data of peripheral blood mononuclear cells (Tirumurugaan et al., 2020). The upregulated ISGs of PPRV infection were different in different cells (Manjunath et al., 2015; Chen et al., 2021). Therefore, it was hypothesized that the IFN-induced ISGs after PPRV infection both has virus and cell specific response. In this study, the upregulated ISG15 and RSAD2 may play an important role in PPRV infection and we already found that both of them could inhibit the viral proliferation (data not shown).

The role of IFNs and ISGs in animal viral control has been investigated. It was found that IFNs expressed by recombinant adenovirus expression vector could delay the clinical signs and virus replication in pigs infected with highly virulent classical swine fever virus, and also provide complete protection to pigs infected with foot-and-mouth disease virus (FMDV) (Chinsangaram et al., 2003; Fernandez-Sainz et al., 2015). Recombinant adenovirus expressing porcine IFN-α and IFN-γ and various siRNAs were found to protect FMDV-infected pigs (Kim et al., 2015). Similarly, the antiviral effects of IFNs and ISGs have also been studied in *Morbillivirus*. Exogenous addition of IFNs inhibited the replication of PPRV and MV (Chang et al., 2019; Aref et al., 2020). RSAD2 inhibited the replication of MV (Kurokawa et al., 2019). Therefore, IFNs and ISGs have the potential to be applied in the veterinary field in the future.

In conclusion, we found that PPRV infects different cell types and could produce different interferon responses, and type III IFNs response are stronger than the type I IFNs responses in EECs. Our study suggested that EECs were an appropriate cell model for PPRV, which can be used for future studies on the mechanism of viral infection and innate immunity.

## Data Availability Statement

The raw data supporting the conclusions of this article will be made available by the authors, without undue reservation.

## Author Contributions

Conceptualization, JT, GL, and CM. Methodology, JT and AT. Experimental work, JT, AT, and HD. Data analysis, HD, NJ and JZ. Writing—original draft preparation, JT. Writing—review and editing, CL, CM, and GL. Supervision, GL, CM, and CL. Funding acquisition, GL, CM, and JZ. All authors have read and agreed to the published version of the manuscript.

## Funding

This study was supported by the National Natural Science Foundation of China (No. 32172832, No. 32000109), Shanghai Sailing Program (20YF1457700), the China Postdoctoral Science Foundation (No. 2019M660885, No. 2021T140718), and the Central Public-interest Scientific Institution Basal Research Fund (2021JB08).

## Conflict of Interest

The authors declare that the research was conducted in the absence of any commercial or financial relationships that could be construed as a potential conflict of interest.

## Publisher’s Note

All claims expressed in this article are solely those of the authors and do not necessarily represent those of their affiliated organizations, or those of the publisher, the editors and the reviewers. Any product that may be evaluated in this article, or claim that may be made by its manufacturer, is not guaranteed or endorsed by the publisher.

## Supplementary Material

Supplementary 1 Cytopathic effects of PPRV in CSFs, GFs, and EECs. CSFs, GFs, and EECs were infected with PPRV, and cytopathic effects were observed at 12, 24, 36, 48, 72, 96, and 120 h post-infection, respectively. Cells not inoculated with PPRV were used as negative controls.
