# Supplementary figures and images for: Peste des Petits Ruminants Virus Exhibits Cell-Dependent Interferon Active Response
Source: Front Cell Infect Microbiol. 2022 May 31;12:874936. doi: 10.3389/fcimb.2022.874936 (PMC9195304; doi:10.3389/fcimb.2022.874936)

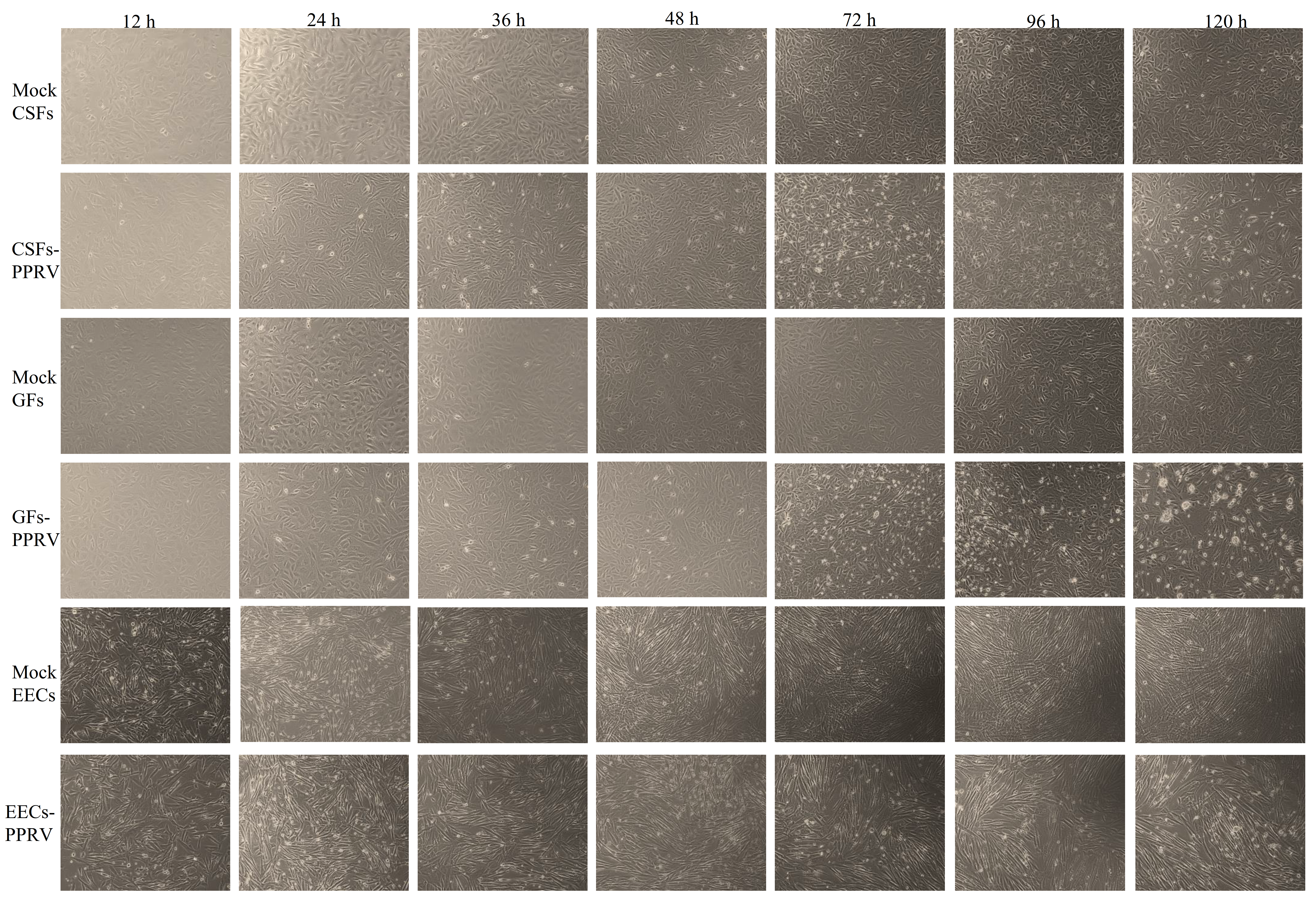

Supplement: Supplementary 1 — Cytopathic effects of PPRV in CSFs, GFs, and EECs. CSFs, GFs, and EECs were infected with PPRV, and cytopathic effects were observed at 12, 24, 36, 48, 72, 96, and 120 h post-infection, respectively. Cells not inoculated with PPRV were used as negative controls. [file Image_1.tif]
